# Supplementary material for: A cascade of care analysis on the elimination of hepatitis C from public hospitals in Madrid
Source: Commun Med (Lond). 2022 Feb 24;2:20. doi: 10.1038/s43856-022-00077-9 (PMC9053180; doi:10.1038/s43856-022-00077-9)
Supplement: Supplementary file 1 — Description of Additional Supplementary Files [file 43856_2022_77_MOESM1_ESM.pdf]

Description of Additional Supplementary Files

File Name: Supplementary Data

Description: Compiled data for Madrid HCV micro-elimination in high-risk groups.
