# Supplementary material for: A cascade of care analysis on the elimination of hepatitis C from public hospitals in Madrid
Source: Commun Med (Lond). 2022 Feb 24;2:20. doi: 10.1038/s43856-022-00077-9 (PMC9053180; doi:10.1038/s43856-022-00077-9)
Supplement: Supplementary file 4 — Reporting Summary [file 43856_2022_77_MOESM4_ESM.pdf]

nature research

Corresponding author(s): DBPR COMMSMED-21-0454

Last updated by author(s): Dec 10, 2021

# Reporting Summary

Nature Research wishes to improve the reproducibility of the work that we publish. This form provides structure for consistency and transparency in reporting. For further information on Nature Research policies, see our Editorial Policies and the Editorial Policy Checklist.

## Statistics

For all statistical analyses, confirm that the following items are present in the figure legend, table legend, main text, or Methods section.

| n/a | Confirmed | |
|---|---|---|
| ☐ | ☒ | The exact sample size ($n$) for each experimental group/condition, given as a discrete number and unit of measurement |
| ☐ | ☒ | A statement on whether measurements were taken from distinct samples or whether the same sample was measured repeatedly |
| ☐ | ☒ | The statistical test(s) used AND whether they are one- or two-sided<br>*Only common tests should be described solely by name; describe more complex techniques in the Methods section.* |
| ☐ | ☒ | A description of all covariates tested |
| ☒ | ☐ | A description of any assumptions or corrections, such as tests of normality and adjustment for multiple comparisons |
| ☐ | ☒ | A full description of the statistical parameters including central tendency (e.g. means) or other basic estimates (e.g. regression coefficient) AND variation (e.g. standard deviation) or associated estimates of uncertainty (e.g. confidence intervals) |
| ☒ | ☐ | For null hypothesis testing, the test statistic (e.g. $F$, $t$, $r$) with confidence intervals, effect sizes, degrees of freedom and $P$ value noted<br>*Give P values as exact values whenever suitable.* |
| ☒ | ☐ | For Bayesian analysis, information on the choice of priors and Markov chain Monte Carlo settings |
| ☒ | ☐ | For hierarchical and complex designs, identification of the appropriate level for tests and full reporting of outcomes |
| ☒ | ☐ | Estimates of effect sizes (e.g. Cohen's $d$, Pearson's $r$), indicating how they were calculated |

*Our web collection on statistics for biologists contains articles on many of the points above.*

## Software and code

Policy information about availability of computer code

| | |
|---|---|
| Data collection | Microsoft Excel |
| Data analysis | Stata SE version 16 |

For manuscripts utilizing custom algorithms or software that are central to the research but not yet described in published literature, software must be made available to editors and reviewers. We strongly encourage code deposition in a community repository (e.g. GitHub). See the Nature Research guidelines for submitting code & software for further information.

## Data

Policy information about availability of data

All manuscripts must include a data availability statement. This statement should provide the following information, where applicable:

- Accession codes, unique identifiers, or web links for publicly available datasets
- A list of figures that have associated raw data
- A description of any restrictions on data availability

Data are available from the corresponding author upon request.

# Field-specific reporting

Please select the one below that is the best fit for your research. If you are not sure, read the appropriate sections before making your selection.

☒ Life sciences ☐ Behavioural & social sciences ☐ Ecological, evolutionary & environmental sciences

For a reference copy of the document with all sections, see nature.com/documents/nr-reporting-summary-flat.pdf

# Life sciences study design

All studies must disclose on these points even when the disclosure is negative.

| | |
|---|---|
| Sample size | Six public hospitals in Madrid were included, covering a population of more than two million people. Assuming a total population of 6,000 patients (in all target groups) across all six hospitals, a sample size of 546 was needed in order to be 95% confident with a precision of +/- 4 degrees. |
| Data exclusions | No data were excluded from the analyses. |
| Replication | Given the nature of this cross-sectional study, findings cannot be reproduced. |
| Randomization | Given this was not a randomized controlled trial, randomization was not relevant to this study. |
| Blinding | Given this was not a randomized controlled trial, blinding was not relevant to this study. |

# Reporting for specific materials, systems and methods

We require information from authors about some types of materials, experimental systems and methods used in many studies. Here, indicate whether each material, system or method listed is relevant to your study. If you are not sure if a list item applies to your research, read the appropriate section before selecting a response.

## Materials & experimental systems

| n/a | Involved in the study | |
|---|---|---|
| ☒ | ☐ | Antibodies |
| ☒ | ☐ | Eukaryotic cell lines |
| ☒ | ☐ | Palaeontology and archaeology |
| ☒ | ☐ | Animals and other organisms |
| ☐ | ☒ | Human research participants |
| ☒ | ☐ | Clinical data |
| ☒ | ☐ | Dual use research of concern |

## Methods

| n/a | Involved in the study | |
|---|---|---|
| ☒ | ☐ | ChIP-seq |
| ☒ | ☐ | Flow cytometry |
| ☒ | ☐ | MRI-based neuroimaging |

## Human research participants

Policy information about studies involving human research participants

| | |
|---|---|
| Population characteristics | In total, there were 3,994 patients across all high-risk groups (125 of whom had two or more comorbidities) and 68.8% were male, 87.2% of Spanish origin, and the mean age was 57.6 years (SD: 14.8). |
| Recruitment | From September 2019 to March 2021, anonymised data were collected in Microsoft Excel through a registry review of all adult patients (18 years or older) in haemodialysis or pre-dialysis programmes, co-infected with HIV, with advanced liver disease, with hereditary haematological diseases, and with transplants, and of people who inject drugs. Six public hospitals in Madrid were included, covering a population of more than two million people. |
| Ethics oversight | Ethical Committee of the Hospital Clínic, Barcelona, Spain. |

Note that full information on the approval of the study protocol must also be provided in the manuscript.
